# Evaluation of different diagnostic methods for spinal tuberculosis infection

**DOI:** 10.1186/s12879-023-08655-5

**Published:** 2023-10-18

**Authors:** Zhaoxin Li, Jin Wang, Xin Xiu, Zhenpeng Shi, Qiang Zhang, Deqiang Chen

**Affiliations:** 1 Shandong University of Traditional Chinese Medicine, Shandong, China; 2 Department of Spinal Orthopedics, Shandong Provincial Hospital of Traditional Chinese Medicine, Shandong, China; 3 Department of Orthopedics, Shandong Public Health Clinical Center, Shandong, China

**Keywords:** Diagnosis, Spinal tuberculosis, Mycobacterium tuberculosis, Xpert MTB/RIF, Metagenomic next-generation sequencing

## Abstract

**Background and purpose:**

Tuberculosis (TB) is the most fatal infectious disease worldwide. Approximately 24.6% of tuberculosis cases are extrapulmonary and predominantly affect the spine. It is difficult to diagnose spinal TB (STB). We aimed to evaluate the diagnostic performance of the Mycobacteria Growth Indicator Tube (MGIT)-960 culture, T-SPOT.TB, Xpert *Mycobacterium tuberculosis* complex (MTB)/resistance to rifampin (RIF), and Metagenomic Next-Generation Sequencing (mNGS) to detect STB.

**Methods:**

We assessed 126 patients presumed to have STB using these four methods. The sensitivity, specificity, positive predictive value (PPV), and negative predictive value (NPV) were calculated using clinical diagnosis as a reference.

**Results:**

Of the patients, 41 were diagnosed with STB and 85 with non-STB. In the STB group, the sensitivity, specificity, PPV, and NPV of the MGIT-960 culture were 29.3% (12/41), 100% (85/85), 100% (12/12), and 74.6% (85/114), respectively. The sensitivity, specificity, PPV, and NPV of T-SPOT.TB were 92.7% (38/41), 82.4% (70/85), 58.5% (31/53), and 95.9% (70/73), respectively. The sensitivity, specificity, PPV, and NPV of the Xpert MTB/RIF assay were 53.7% (22/41), 100% (85/85), 100% (22/22), and 81.7% (85/104), respectively. The sensitivity, specificity, PPV, and NPV of mNGS were 39.0% (16/41), 98.8% (84/85), 94.1% (16/17), and 77.1% (84/109), respectively. The sensitivity, specificity, PPV, and NPV of mNGS + Xpert MTB/RIF were 73.2% (30/41), 100% (85/85), 96.8% (30/31), and 72.0% (85/118), respectively. The sensitivity, specificity, PPV, and NPV of the mNGS + T-spot assay were 97.6% (40/41), 100% (85/85), 67.9% (38/56), and 75.9% (85/113), respectively. Moreover, the sensitivity, specificity, PPV, and NPV of T-spot + Xpert MTB/RIF were 95.1% (39/41), 100% (85/85), 72.2% (39/54), and 81.0% (85/105), respectively.

**Conclusions:**

T-SPOT.TB is the most effective method for diagnosing STB; however, Xpert MTB/RIF is more reliable and can detect RIF resistance. Clinicians can use mNGS to identify pathogens in patients with spinal infections; these pathogens appeared to be more meaningful in guiding the clinical management of patients in the non-STB group. The combination of Xpert MTB/RIF and mNGS can improve the early diagnosis rate and drug resistance detection, reduce the diagnostic cycle, and provide early targeted anti-TB treatment for patients with STB.

**Supplementary Information:**

The online version contains supplementary material available at 10.1186/s12879-023-08655-5.

## Introduction

According to a 2022 report by the World Health Organization, approximately 6.4 million people will have TB worldwide. More than 1.4 million people died of TB in 2021, making it the second leading cause of death from a single source of infection after COVID-19; China has a high burden of TB (WHO, 2022) [1]. Of the patients with tuberculosis, 24.6% have extrapulmonary tuberculosis, of which 9.8% have STB [2]. STB accounts for approximately half of the musculoskeletal tuberculosis cases [3]. Lumbar tuberculosis is the most common manifestation of STB, followed by thoracic tuberculosis. The incidence of STB is increasing worldwide [4].

Severe STB can lead to spinal deformities and even paraplegia, and early identification and diagnosis are central to reducing the STB-associated disability rates and mortality [5]. Delayed initial diagnosis and STB confirmation are attributed to the prolonged and inferior treatment process. Furthermore, drug-resistant tuberculosis is predominant in China. Tuberculosis culture is the gold standard for the clinical diagnosis of TB. However, the culture cycle is prolonged and cannot detect simultaneous pathogen infection [6]. Immunological tests, such as the T-SPOT.TB, can detect previous infections in recovered patients. Although T-SPOT.TB has good sensitivity, it has poor specificity [7] [8]. Xpert MTB/ RIF is a rapid and reliable diagnostic method for both tuberculosis and rifampicin resistance. The early application of Xpert MTB/RIF can reduce RIF resistance effectively [9]. mNGS offers advantages in the detection of clinical samples, without prior suspicion of certain pathogens. In addition, mNGS can improve pathogen detection associated with extrapulmonary infections, with potential advantages in speed and sensitivity [10]. mNGS and Xpert MTB/RIF offer significant advantages over the traditional MTB detection methods [11].

However, few reports have elucidated the rapid diagnosis of suspected STB. To further evaluate the diagnostic ability of mNGS for suspected STB, we aimed to evaluate the diagnostic performance of mNGS, Xpert MTB/RIF, T-SPOT.TB, and MGIT-960 culture in detecting STB. We analyzed the diagnostic value of Xpert MTB/RIF and mNGS in the identification of spinal infection pathogens and explored the application of mNGS and Xpert MTB/RIF combined with rapid diagnostic tests in the clinical treatment.

## Materials and methods

### Patient enrollment

From May 2020 and October 2020, 126 patients with clinical manifestations of suspected STB were selected from the Shandong Public Health Clinical Center. They signed an informed consent form. The inclusion criteria were as follows: (1) persistent pain in the spine for at least 3 weeks; (2) fever; (3) abnormalities on spinal MRI, suggesting possible spinal infection; and (4) abnormal erythrocyte sedimentation rate.

### Diagnostic criteria

A comprehensive reference standard was used to confirm the diagnosis of STB. The criteria included clinical symptoms and laboratory, pathologic, and imaging tests. Clinical diagnostic criteria included clinical symptoms and imaging tests consistent with STB. Pathologic diagnosis included inflammatory granulomas, caseous necrosis, or dead bone and cavities were found in the lesions. Microbiologic examination: ll patients received mNGS, Xpert MTB/RIF, T-SPOT.TB and MGIT-960 tests. Patients were diagnosed with STB when the mNGS or Xpert MTB/RIF or MGIT-960 test result was positive for Mycobacterium tuberculosis. Some patients only had a positive test result for T-SPOT.TB, but we took into consideration the possibility of false-positive test results for T-SPOT.TB. Therefore, anti-tuberculosis treatment was administered to these patients and those in whom anti-tuberculosis treatment was effective, were diagnosed with STB. Microbiological examination is direct evidence for STB diagnosis, and serologic examination and imaging examination are important supplements for the diagnosis of STB.

### Sample collection and processing

All clinical samples were pre-treatment samples and included serum, pus, and pathological tissues. Pus and pathological tissues were collected from the suspected infection sites. Lesion tissue specimens were obtained via percutaneous puncture of the spinal lesions under the guidance of a C-arm X-ray machine. We determined the sample volumes and preparations according to the requirements of each test. All samples were immediately sent for MGIT-960 and bacterial culture based on the manufacturer’s instructions.

### MGIT-960 culture

The MGIT-960 culture was prepared according to the manufacturer’s instructions. Pus specimens were purified and diluted by treating them with equal volumes of 2% sodium hydroxide and 0.5% N-acetyl-1-cysteine-sodium hydroxide for 15 min. The final pellet was resuspended in 1 ml of phosphate buffer to provide a sufficient sample volume for the liquid culture for up to 42 days in an MGIT-960 system (Becton Dickinson) with continuous sample monitoring. The results were reported according to the manufacturer’s instructions. Positive tubes were observed under a microscope. Standard drug susceptibility testing and RIF tests were performed for the positive cultures using an MGIT-960 IR kit (Becton Dickinson), according to the manufacturer’s instructions.

### T-SPOT.TB

Mononuclear cells were isolated from 5 ml of peripheral venous blood samples and assayed using a T-SPOT.TB assay kit (Oxford Immunotec Ltd., Abingdon, UK). The cell suspension was seeded onto a T-SPOT column. TB plates were incubated with 6-kDa Early Secreted Antigenic Target, 10-kDa culture filtrate protein, or the positive control. We added 100 µl of cell suspension into the corresponding microwells. They were cultured in an incubator with 5% CO2 at 37 °C. The microwells were washed thrice with phosphate-buffered saline. We added 50 µl of the secondary antibody into each well for 1 h of incubation. Subsequently, the wells were washed and 50 µl of substrate solution was added into them. The plate was processed under light avoidance for 7 min before termination with distilled water. The number of spots was measured.

### Xpert MTB/RIF ASSAY

Tissue specimens collected by surgery or puncture were cut into 2 to 3 mm pieces. We added 2 ml of the sample reagent buffer containing NaOH and isopropanol in a ratio of 3:1, incubated for 15 min at room temperature, and grounded thoroughly until a homogeneous suspension was obtained. The samples were assessed by Xpert (Cepheid, Sunnyvale, CA, USA) according to the manufacturer’s instructions, mixed with 1 ml of pus sample with 2 ml of Xpert sample reagent, vortexed for at least 10 s, and incubated at room temperature for 10 min. They were vortexed again for 10 s and incubated for 5 min at room temperature. We transferred 2 ml of the mixture into an Xpert cylinder and loaded it into a GeneXpert instrument to record the results.

### mNGS analysis

DNA was extracted from the clinical specimens using a TIANamp Micro DNA Kit (DP316, TIANGEN BIOTECH) according to the manufacturer’s instructions. Sequencing libraries were constructed and sequenced using an Illumina MiSeq instrument. The reads were analyzed using sequence-based ultra-rapid pathogen identification, which first identifies and subtracts the human host sequences. Microbial genome databases were used to classify the remaining data. The classification reference databases were downloaded from NCBI (http://www.ftp.ncbi.nlm.nih.gov/genomes/). The infectious pathogen was identified using at least 50 unique reads from a single species of bacteria, viruses, fungi, or parasites.

### Statistical analysis

A clinical characteristics analysis table was prepared to collect the demographic characteristics and clinical data of the cases. The data include the sex, age, and lesion location. SPSS software version 26.0 was used for the statistical analysis. Statistical differences were compared by the Chi-square test, corrected chi-square test, and Fisher exact test for categorical variables. The continuous variables were statistically differentiated using the t-test and described as mean ± standard deviation.

## Results

### Study patients

We enrolled 126 patients with suspected spinal infections in this retrospective study. Finally, 41 patients were diagnosed with STB and 85 with non-STB. The mean age of STB group was 52.63 ± 18.82 years; it comprised 20 men and 21 women. The mean age of the non-STB group was 53.92 ± 15.33 years; it comprised 56 men and 29 women. The differences in sex and age between the groups were statistically insignificant. Table 1 summarizes the clinical features of the groups.

**Table 1:** Clinical characteristics of the studied patients

Clinical characteristics	Spinal STB infection	Non-STB spinal infection	p value of STB vs. non-STB
Age, years (mean ± SD)	52.63 ± 18.82	53.92 ± 15.33	P = 0.684
Gender,n(%)			
Male	20(48.4%)	56(65.9%)	X^2^ = 3.38; p = 0.07
Female	21(51.2)	29(34.1%)	
Site of spine lesion, n (%)			
Cervical vertebra	3(7.3%)	2(2.3%)	
Cervithoracic spine	1(2.4%)	12(14.1%)	
Thoracic vertebra	8(19.5%)	0(0.0%)	
Lumbar vertebra	29(70.7%)	71(83.5%)	

n: number of patients, STB: Spinal tuberculosis, non-STB: non-spinal tuberculosis

### Pathogen composition

**Fig. 1:**
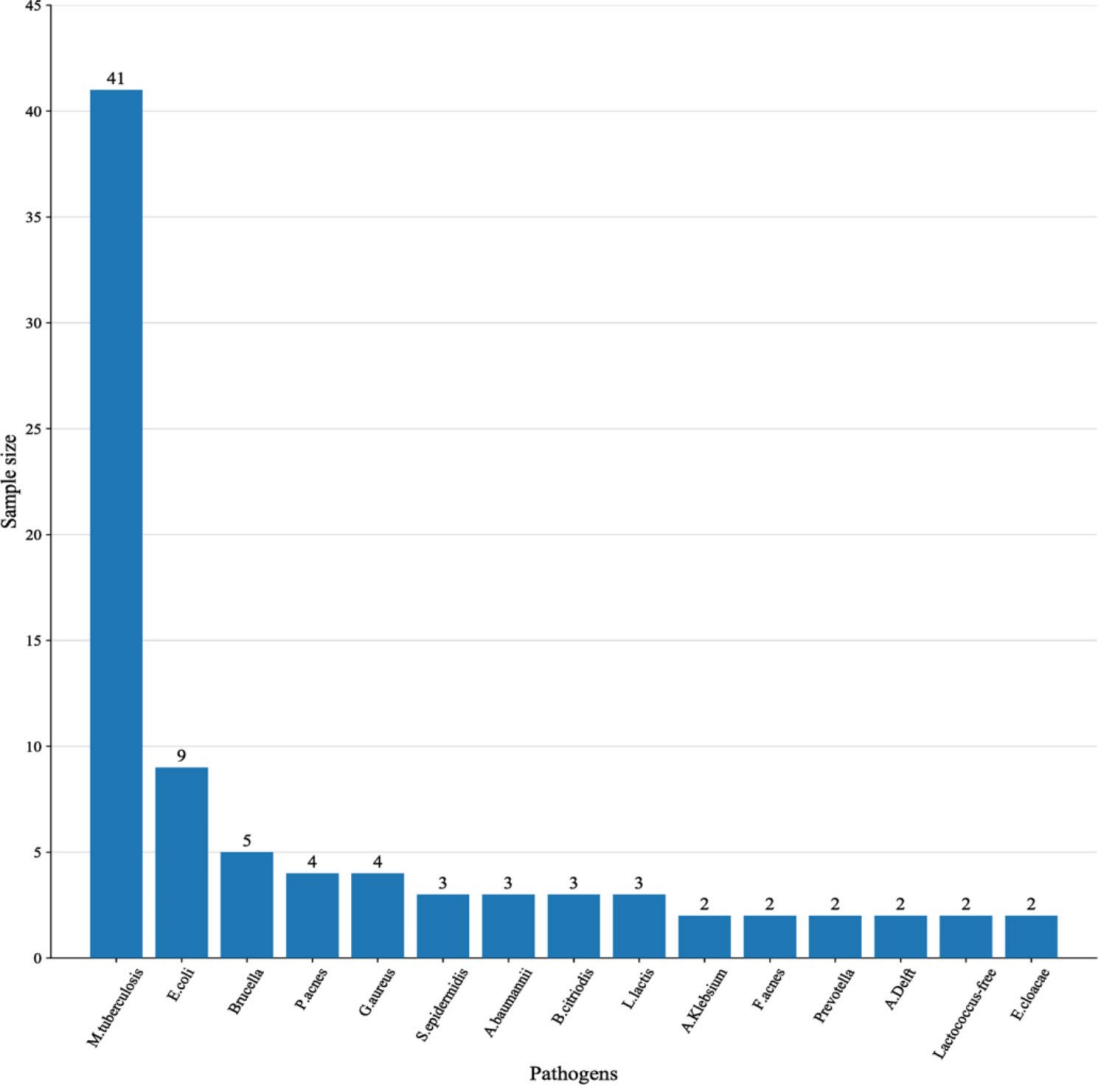
87 patients with detectable pathogenic pathogens

Four modalities, namely MGIT-960 culture, T-SPOT.TB, Xpert MTB/RIF, and mNGS, were used to assess 126 patients with spinal infections. In the STB group, 16 samples were detected for TB by mNGS, of which 10 samples were detected for other pathogens as well. In the non-STB group, 77 samples were detected for pathogens by mNGS, of which 38 were detected only once. Supplementary table S1 enlists the pathogens from all the samples. Figure 1 depicts the sample size for each pathogen.

### Diagnostic performance of mNGS and other methods in patients with STB

In the STB group, the sensitivity, specificity, PPV, and NPV of the MGIT-960 culture were 29.3% (12/41), 100% (85/85), 100% (12/12), and 74.6% (85/114), respectively. Of the 12 patients with positive MGIT-960 cultures, 7 were sensitive to RIF (58.3%). The MGIT-960 culture demonstrated lower sensitivity. The sensitivity, specificity, PPV, and NPV of T-SPOT.TB were 92.7% (38/41), 82.4% (70/85), 58.5% (31/53), and 95.9% (70/73), respectively. Xpert MTB/RIF detected 22 positive samples and 9 RIF-sensitive samples. The sensitivity, specificity, PPV and NPV of Xpert MTB/RIF were 53.7% (22/41), 100% (85/85), 100% (22/22), and 81.7% (85/104), respectively. The sensitivity, specificity, PPV, and NPV of mNGS were 39.0% (16/41), 98.8% (84/85), 94.1% (16/17), and 77.1% (84/109), respectively. Thirteen patients were sensitive to RIF in all STB patients, nine in Xpert MTB/RIF, and seven in MGIT 960 culture. Three patients were assessed using both the Xpert MTB/RIF and MGIT 960 cultures. The remaining patients were not sensitive to RIF. The sensitivity difference between mNGS and T-SPOT.TB was statistically significant (χ^2^ = 23.92, p = 0.00). The difference between mNGS and Xpert MTB/RIF and mNGS and MGIT-960 cultures was statistically insignificant (χ^2^ = 1.77, p = 0.18; χ^2^ = 0.87, p = 0.35). The specificity difference between mNGS and T-spot was statistically significant (χ^2^ = 11.66, p = 0.001). The specificity difference between mNGS and Xpert MTB/RIF and mNGS and MGIT-960 cultures was statistically insignificant (p = 1.00; p = 1.00) (Table 2).

**Table 2:** Performance of different methods for diagnosis of STB

Methods	Sensitivity (%,N,95% CI)	Specificity(%, N, 95% CI)	PPV (%,N,95% CI)	NPV (%,N,95% CI)	p value Sensitivity	p value Specificity
mNGS	39.0%,(16/41),24.2-55.5%	98.8%,(84/85),0.00-6.4%	94.1%,(16/17),71.3-99.9%	77.1%,(84/109),68.0-84.6%		
MGIT-960 culture	29.3%,(12/41),16.1-45.5%	100%,(85/85),95.8-100%	100%,(12/12),73.5-100%	74.6,(85/114),65.6-82.3%	χ^2^ = 0.87, p = 0.35a	p = 1.00a
T-SPOT.TB	92.7%,(38/41),80.1-98.5%	82.4%,(70/85),10.2-27.4%	58.5%,(31/53), 44.1-71.9%	95.9%,(70/73),88.5-99.1%	χ^2^ = 23.92,p = 0.00^b^	χ^2^ = 11.66,p = 0.001^b^
Xpert MTB/RIF	53.7%,(22/41),37.4-69.3%	100%,(85/85), 95.8-100%	100%,(22/22),84.6-100%	81.7%,(85/104),72.9-88.6%	χ^2^ = 1.77,p = 0.18^c^	p = 1.00^c^

N: number of patients, CI: confidence interval, ^a^mNGS vs. MGIT-960 culture, ^b^mNGS vs. T-SPOT.TB, ^c^mNGS vs. Xpert MTB/RIF

N: number of patients, CI: confidence interval, ^a^mNGS vs. MGIT-960 culture, ^b^mNGS vs. T-SPOT.TB, ^c^mNGS vs. Xpert MTB/RIF

### Diagnostic performance of combined diagnostic assays in patients with STB

To obtain accurate diagnostic results rapidly, we combined mNGS, Xpert MTB/RIF, and T-SPOT.TB for the analysis. In the STB group, the sensitivity, specificity, PPV, and NPV of mNGS + Xpert MTB/RIF were 73.2% (30/41), 100% (85/85), 96.8% (30/31), and 72.0% (85/118), respectively. The sensitivity, specificity, PPV, and NPV of the mNGS + T-SPOT.TB assays were 97.6% (40/41), 100% (85/85), 67.9% (38/56), and 75.9% (85/113), respectively. The sensitivity, specificity, PPV, and NPV of the T-SPOT.TB + Xpert MTB/RIF assays were 95.1% (39/41), 100% (85/85), 72.2% (39/54), and 81.0% (85/105), respectively. Sensitivity differences between the mNGS + Xpert MTB/RIF group and the mNGS + T-spot group and the T-spot + Xpert MTB/RIF group were statistically significant (χ^2^ = 7.91, p = 0.005; χ^2^ = 5.85, p = 0.016). mNGS detected MTB in 8 Xpert MTB/RIF-negative patients and in 2 T-SPOT.TB-negative patients The difference in PPV between MTB/RIF group was statistically significant (χ^2^ = 8.15, p = 0.004; χ^2^ = 6.25, p = 0.012) (Table 3).

**Table 3:** Performance of combined diagnostic assays for diagnosis of STB

Methods	Sensitivity (%,N,95% CI)	Specificity(%,N,95% CI)	PPV (%,N,95% CI)	NPV (%,N,95% CI)	p value Sensitivity	p value PPV
mNGS + Xpert MTB/RIF	73.2%,(30/41),57.1-85.5%	100%,(85/85),95.8-100%	96.8%,(30/31),83.3-99.9%	72.0%,(85/118),63.0-79.9%		
mNGS + T-spot	97.6%,(40/41),87.1-99.9%	100%,(85/85),95.8-100%	67.9%,(38/56),54.0-79.7%	75.9%,(85/113),66.2-82.0%	χ^2^ = 7.91, p = 0.005^d^	χ^2^ = 8.15, p = 0.004^d^
T-spot + Xpert MTB/RIF	95.1%,(39/41),83.5-99.4%	100%,(85/85),95.8-100%	72.2%,(39/54),58.4-83.5%	81.0%,(85/105),72.1-88.0%	χ^2^ = 5.85, p = 0.016^e^	χ^2^ = 6.25, p = 0.012^e^

N: number of patients, CI: confidence interval, ^d^mNGS+Xpert MTB/RIF vs. mNGS + T-spot, ^e^mNGS+Xpert MTB/RIF vs. T-spot + Xpert MTB/RIF

## Discussion

An accurate diagnosis of STB is difficult because it is a rare bacterial disease, the specimens are difficult to obtain, and the patients are often treated with relevant medications, further reducing bacterial production [12]. The diagnosis depends primarily on the clinical features, imaging findings, and combined pathogen testing. Delayed diagnosis can lead to neurological symptoms and other serious consequences, such as paraplegia. Conventional detection methods demonstrate low positive detection rates and prolonged detection cycles. The accurate and rapid diagnosis of STB poses a challenge for the clinicians. Traditional detection methods do not meet the requirements of clinical diagnosis.

In this study, we compared four methods for STB detection. Despite cultures being the gold standard for laboratory diagnosis, MGIT-960 cultures reported a low detection rate (29.3%), indicating that the cultures may be less sensitive to STB. Of the 12 patients with positive MGIT-960 cultures, 7 were sensitive to RIF (58.3%). Also in these 7 patients, two of them had drug susceptibility testing results that were streptomycin, rifampicin, isoniazid, ethambutol, pyrazinamide, amikacin, levofloxacin, capreomycin, propylthioisonicotinic acid, and p-amino sulfosalicylic acid sensitivity.Because of the lack of bacterial properties, cultures can lead to misclassification; therefore, it was more appropriate to use the clinical diagnostic results as controls.

T-SPOT.TB is a promising diagnostic tool for STB. The sensitivity (92.7%) of T-SPOT.TB was higher than that reported previously (71.4% and 88.3%) [13] [14]. The sample sizes vary across studies and affect the results. Overall, our results suggested that the sensitivity of T-SPOT.TB has improved. However, its specificity was 82.4%, and the T-SPOT.TB results were negative. T-SPOT.TB results can be considered an important predictor of STB diagnosis, principally because of its low specificity. TB is a prevalent latent infection caused by *Mycobacterium tuberculosis* [15]. In the non-STB group, 15 patients (17.6%) were positive for T-SPOT.TB, and mNGS was used to detect the corresponding bacteria. Thus, the T-SPOT.TB results supposedly comprised false positives.

Xpert MTB/RIF demonstrates good sensitivity (53.7%) and specificity (100%). Of the 22 Xpert MTB/RIF-positive patients in the STB group, 9 were sensitive to RIF (40.9%). Compared with other detection methods, Xpert MTB/RIF detection is more reliable. RIF in TB leads to unnecessary and prolonged treatment. For the patients with suspected STB, Xpert MTB/RIF may help confirm the diagnosis, particularly by detecting RIF. In one prospective study, the Xpert MTB/RIF assay demonstrated a sensitivity and specificity of 95.6% and 96.2%, respectively, for STB, compared with that of a reference standard for liquid-cultured tissues [16]. This finding is of great significance for guiding clinical treatment. The sensitivity of the Xpert MTB/RIF assay differed across extrapulmonary samples. The special anatomical structure of the spine makes it difficult to obtain clinical specimens of STBs, except during surgery [17].

Xpert MTB/RIF demonstrates high sensitivity and specificity and reduces patient retention and resistance [18]. The specimens may contain numerous dead bacteria and neutrophils; however, the Xpert test is less affected by bacterial viability, which can be used as the diagnostic modality for the rapid detection of RIF [9]. One study reported on several false results, particularly for RIF, suggesting possible multidrug resistance cases. Xpert MTB/RIF can only detect RIF; therefore, it should be used as an additional diagnostic test for STB [19].

In the STB group, the sensitivity of mNGS was 39.0%, and the sensitivity difference was statistically insignificant. The specificity of mNGS (98.8%) was lower than that of Xpert MTB/RIF and MGIT-960 culture (100%), and the difference was statistically insignificant. mNGS demonstrated a lower sensitivity for the STB group. However, it had a higher sensitivity (90.6%) for the non-STB group, indicating that the pathogen identified by mNGS is more useful for guiding the clinical treatment of the non-STB group. mNGS is a reliable test for patients with suspected STB, in addition to Xpert MTB/RIF. We detected a Brucella lumbar spine infection in one patient, suggesting the possibility of false-positive mNGS in STB. Furthermore, previous studies have reported on false-positive mNGS results in tests for suspected spinal infections [20] [21].

Compared with other methods, mNGS facilitates the rapid identification of pathogenic microbial infections because its detection time is relatively shorter than that of culture. mNGS analysis typically detects more than one pathogen in a single test, although genes are sometimes extracted from contaminated or background microbes. *Propionibacterium acnes* is the most commonly detected bacterium [22]. Clinicians require a thorough understanding of these infections to distinguish the causative organisms; Brucella infection has a detection of 80%. Brucellosis is a zoonotic disease with high incidence. Similar to STB, spinal brucellosis is one of the most common forms of brucellosis in humans. Brucellosis may be misdiagnosed as TB because its clinical features and basic laboratory parameters are comparable to those of TB [23]. The use of mNGS has improved the diagnosis of invasive fungal infections outside the lungs. Two patients diagnosed with fungal infections were identified using mNGS as having *Aspergillus. fumigatus* and *A. mirabilis*. *A. fumigis* is the primary species detected in Aspergillus [24]. It is difficult to distinguish between multiple pathogens clinically, and the number of patients with suspected STB in hospitals is gradually increasing. mNGS can detect almost all clinical pathogens, provide pathogen information, and even discover new pathogens [21]. Targeted antibiotic therapy based on mNGS has achieved certain results [25]. Moreover, mNGS is less affected by antibiotics [26]. In a smear test of an extrapulmonary tuberculosis sample, mNGS can identify the possible pathogens within 48 h [10]. In previous studies, mNGS performs well in suspected infectious diseases [20] [27]. However, its ability to discriminate between suspected STB has been poorly reported [21].

There are some limitations to mNGS. In this study, mNGS was not sensitive to STB and incurred high costs. MTB is an endobacterium and its detection requires wall breaking to release nucleic acids. Most of the mNGS results on clinical specimens are derived from host cells, and only 0.00001–0.7% of the reads were used successfully for diagnosis. There is a risk of contamination during specimen processing. Furthermore, mNGS detected a bacterium unrelated to lumbar spine infection in the non-STB group, which may be related to skin hair follicles. In addition, it can detect DNA and RNA pathogens; however, RNA is unstable and degrades easily. Therefore, clinicians should increase the sequencing depth and filter out the host reads, besides carefully interpreting the results [12].

We can use Xpert MTB/RIF and mNGS in combination to improve the sensitivity. The sensitivity (73.2%) of mNGS + Xpert MTB/RIF was lower than that of the remaining two combinations, whereas the PPV (96.8%) was significant. The probability of false positives is significantly reduced. mNGS could be used to improve the sensitivity and timely diagnosis of TBM when combined with Xpert or traditional diagnostic tests [28].

This study has some limitations. First, the STB sample size was relatively small; therefore, researchers should increase the number of positive samples in future studies. In addition, we did not include healthy controls. Finally, the processing method for the mNGS samples was optimized. Researchers should develop standardized procedures in future studies to improve the detection efficiency.

## Conclusions

Of the four detection methods, T-SPOT.TB is the most effective technique for diagnosing STB. Nonetheless, Xpert MTB/RIF is more reliable, can detect RIF, and be used to identify pathogens in patients with spinal infection. The pathogens identified by mNGS appear to be more meaningful in guiding clinical management in the non-STB group. The Xpert MTB/RIF + mNGS combination improves the early diagnosis of STB and detection of drug resistance. Furthermore, it reduces the diagnostic cycle and initiates early targeted anti-TB treatment.

### Electronic supplementary material

Below is the link to the electronic supplementary material.

Supplementary Material 1

## Data Availability

All data generated or analysed during this study are included in this published article. The data that support the findings of this study are available from the corresponding author upon reasonable request.

## Abbreviations

TB: Tuberculosis
STB: Spinal tuberculosis
mNGS: Metagenomic Next-Generation Sequencin
PPV: positive predictive value
NPV: negative predictive value
EPTB: extrapulmonary tuberculosis
